# PON2 ameliorates Ang II‐induced cardiomyocyte injury by targeting the CANX/NOX4 signaling pathway

**DOI:** 10.1002/iid3.765

**Published:** 2023-02-09

**Authors:** Yuanzheng Ye, Jian Zhang, Yankai Guo, Jiajun Zhu, Baopeng Tang, Ping Fan

**Affiliations:** ^1^ Department of Cardiac Function, State Key Laboratory of Pathogenesis Prevention and Treatment of High Incidence Diseases in Central Asia The First Affiliated Hospital of Xinjiang Medical University Urumqi Xinjiang China; ^2^ Xinjiang Key Laboratory of Cardiac Electrophysiology and Cardiac Remodeling The First Affiliated Hospital of Xinjiang Medical University Urumqi Xinjiang China; ^3^ Cardiact Care Unit The First Affiliated Hospital of Xinjiang Medical University Urumqi Xinjiang China; ^4^ Department of Cardiac Pacing and Electrophysiology The First Affiliated Hospital of Xinjiang Medical University Urumqi Xinjiang China; ^5^ Department of Function Bazhou people's Hospital Korla Xinjiang China

**Keywords:** Ang II‐induced cardiomyocyte injury, *CANX*/*NOX4* signaling pathway, heart failure, PON2

## Abstract

**Background:**

The incidence of heart failure (HF) presents an escalating trend annually, second only to cancer. Few literatures are available regarding on the role of paraoxonase 2 (*PON2*) in HF so far despite the protective role of PON2 in cardiovascular diseases.

**Methods:**

*PON2* expression in AC16 cells was examined with reverse transcriptase‐quantitative polymerase chain reaction and western blot following angiotensin II (Ang II) challenging. After *PON2* elevation, 2, 7‐dichlorofluorescein diacetate assay estimated *reactive oxygen species* content, related kits appraised oxidative stress, enzyme‐linked immunosorbent assay evaluated inflammatory levels, and Western blot was applied to the analysis of apoptosis levels. Research on cytoskeleton was conducted by immunofluorescence (IF), and Western blot analysis of the expressions of hypertrophy‐related proteins was performed. BioGRID and GeneMania databases were used to analyze the relationship between *PON2* and Calnexin (*CANX*), which was corroborated by co‐immunoprecipitation experiment. Subsequently, *PON2* and *CANX* were simultaneously overexpressed in AC16 cells induced by Ang II to further figure out the mechanism.

**Results:**

*PON2* expression was depleted in Ang II‐induced AC16 cells. *PON2* might mediate *CANX/NOX4* signaling to inhibit oxidation, inflammatory, hypertrophy, and damage in Ang II‐induced AC16 cells.

**Conclusion:**

*PON2* can ease Ang II‐induced cardiomyocyte injury via targeting *CANX/NOX4* signaling.

## Graphical abstract

The incidence of heart failure (HF) is increasing year by year, second only to cancer. Paraoxonase 2 (PON2) plays a protective role in cardiovascular diseases, but the role of PON2 in HF has not been reported so far. In this study, we concluded that PON2 can ameliorate Ang II‐induced cardiomyocyte injury by targeting the Calnexin (CANX)/NOX4 signaling pathway.

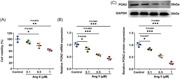

## 1 INTRODUCTION

Heart failure (HF) is a clinical syndrome on account of impaired ventricular filling and/or ejection function attributed to structural and functional heart defects.^1^ Over 26,000,000 people are estimated to suffer from HF globally, and its incidence presents an escalating trend annually.^2^ With the alternations in people's lifestyle and living environment, the prevalence of HF is ascending second only to tumor. Therefore, reasonable and effective prevention and treatment of HF is the current problem to solve.

As an antioxidant enzyme, Paraoxonase 2 (*PON2*) belongs to detoxifying lactase family.^3^ A previous study has found that *PON2* has antioxidant and atherosclerotic protective effects in cardiovascular diseases.^4^ *PON2* can prevent acute myocardial ischemia‐reperfusion injury by regulating mitochondrial function and oxidative stress through the PI3K/Akt/GSK‐3β RISK pathway.^5^ Moreover, *PON2* deficiency significantly exacerbates transverse aortic coarctation‐elicited myocardial fibrosis, left ventricular remodeling as well as oxidative stress.^6^ These findings imply that *PON2* elicit protective effect on HF. In addition, the renin‐angiotensin‐aldosterone system (RAAS) plays a key role in regulating blood pressure and volume homeostasis in the process of HF,^7^ while angiotensin II (Ang II) is a core component of the renin‐angiotensin‐angiotensin system (RAS) and plays a key role in the occurrence and development of cardiac remodeling.^8^ It has shown that the expression of *PON2* is decreased in Ang II‐induced vascular smooth muscle cells and hypertensive rat vascular tissues, and *PON2* can be activated by Fisetin to produce antioxidant effects.^9^ Therefore, it is reasonable to speculate that *PON2* may also be involved in the protection of Ang II‐induced myocardial cell damage.

*PON2* may target Calnexin (*CANX*) predicted by BioGrid and GeneMania databases. Study has found that *CANX* can indirectly affect SERCA (Sarco/endoplasmic reticulum Ca(2+)‐transport ATPase) activity, and then lead to dysregulation of calcium, hence participating in the process of HF.^10^ Moreover, *CANX* is a *NOX4* interaction protein, and reduction of *CANX* can reduce *NOX4* expression and reactive oxygen species (*ROS*) formation.^11^ Meanwhile, *PON2* elevation can decline *NOX4* expression.^12^ Nevertheless, few literatures are available regarding on the role of *PON2* and *CANX/NOX4* in HF.

Therefore, in this paper, we hypothesized that *PON2* ameliorates Ang II‐induced cardiomyocyte injury by targeting the *CANX/NOX4* signaling pathway. Our experiment might lay a theoretical foundation for the clinical treatment of HF.

## 2 MATERIALS AND METHODS

### 2.1 Database

BioGRID (https://thebiogrid.org/) and GeneMania (https://genemania.org/) databases were used to analyze the possible interaction between *PON2* and *CANX*.

### 2.2 Cell culture

Human cardiomyocyte‐like cells AC16 purchased from BeNa Culture Collection (BNCC339980) were cultured in DMEM (Gibco; Thermo Fisher Scientific) containing 10% fetal bovine serum (Gibco; Thermo Fisher Scientific) in 5% CO_2_ at 37°C. The effect of Ang II on AC16 cells was assessed with 0.1, 0.5, and 1 μM human Ang II (HY‐13948; MedChem Express) for 24 h.^13^

#### 2.2.1 Cell‐counting‐Kit‐8

The AC16 cells were seeded in 96‐well plates. After the cells were treated with Ang II for 24 h, CCK‐8 liquid was added to each well for 2 h of incubation according to the manufacturer's instructions. The absorbance in each well was measured with a microplate reader at 450 nm.

### 2.3 Quantitative reverse transcriptase polymerase chain reaction (qRT‐PCR)

The spectroscopy was adopted to detect the concentration and purity of the RNA samples isolated from cells with TRIzol Reagent (Invitrogen). Reverse transcription of RNA to complementary DNA were performed with quantiTect Reverse Transcription Kit (Qiagen). Next, PCR amplification was conducted with TB Green® Premix ExTaqII (Takara). β‐actin was utilized to normalize the mRNA expression levels.

### 2.4 Western blot

The protein extractions were obtained from AC16 cells with RIPA lysate and the protein concentrations detected by BCAKit (P0010; Beyotime). Polyvinylidene fluoride membranes were to move the polyacrylamide gel electrophoresis‐separated proteins (30 μg/well), before the supplementation of previously indicated primary antibodies (1:1000) and secondary antibodies (1:5000). Members were tracked with the ECL Plus Western blot analysis Detection System (GE Healthcare), followed by analysis of image J software.

### 2.5 Cell transfection

*CANX* and *PON2* overexpression vector (Oe‐*CANX*; Oe‐*PON2*) and their empty vector (Oe‐NC) were purchased from Shanghai GeneChem Co. Lipofectamine 2000 (Invitrogen) was applied to plasmid transduction.

### 2.6 Measurement of reactive oxygen species

To determine the ROS levels, diluted 2, 7‐dichlorofluorescein diacetate (DCFH‐DA) (10 µM) was to cultivate treated AC16 cells for 20 min protected from light. Then a confocal microscope (Olympus FluoView FV1000) was to record the fluorescence intensity.

### 2.7 Co‐immunoprecipitation (Co‐IP)

Following the lysis of AC16 cells in Tris/HCl, pH 7.5, 1% Triton. Appropriate antibody (2 μg) and Protein A/G‐Sepharose beads (GE Healthcare) were added to the supernatant, respectively, for 1.5 h. Western blot was employed for analysis after the beads were rinsed in lysis buffer and cultivated with Laemmli buffer for 5 min at 95°C.

### 2.8 Enzyme‐linked immunosorbent assay (ELISA)

*TNF‐α, IL‐1β, IL‐6, malonaldehyde (MDA)*, and *superoxide dismutase* (*SOD*) content were measured in the supernatant of treated cells, using relevant ELISA kits, respectively, in light of the manual provided by the manufacturer.

### 2.9 Immunofluorescence assay

For the cytoskeletal assay, immobilization and permeation in treated cells (3 × 10^3^ cells/well) were, respectively, carried out with 4% paraformaldehyde and 0.2% Triton X‐100. 0.02% 4', 6‐diaminyl‐2‐phenylindoles was to stain the cells that were probed with 2.5% rhodamine phalloidin for 20 min after blocking in 1% bovine serum albumin for 1 h. Images were analyzed under a fluorescence microscopy with Image‐Pro Plus version 6.0 software.

### 2.10 Statistical analysis

Data analyzed through GraphPad Prism 6 are provided in the format of mean ± SD. Analysis of variance, together with Tukey's post hoc test compared differences among various groups. The threshold of significance was confirmed when *p* < 0.05.

## 3 RESULTS

### 3.1 PON2 expression was declined in Ang II‐challenged AC16 cells

**Figure 1:**
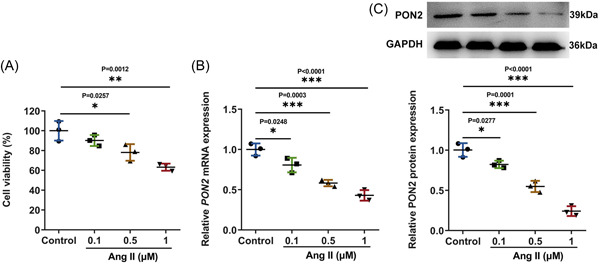
*PON2* expression was declined in Ang II‐challenged AC16 cells. (A) Cell‐counting‐kit‐8 assay was used to detect the cell viability. Analysis of *PON2* expression by RT‐qPCR (B) and Western blot (C). Ang II, angiotensin II; PON2, paraoxonase 2; RT‐qPCR, reverse transcriptase‐quantitative polymerase chain reaction.

Cell‐counting‐kit‐8 results showed that the survival rate of AC16 cells decreased significantly with the increase of Ang II‐induced concentration, and the survival rate of AC16 cells was about 60% when the concentration was 1 μM (Figure 1A,B). Reverse transcriptase‐quantitative polymerase chain reaction (RT‐qPCR) and Western blot were used to detect PON2 expression in AC16 cells challenged with Ang II. The results exposed that PON2 expression was declined with the ascending doses of Ang II relative to the control group (Figure 1B,C). Since PON2 expression is the most prominently decreased when treated by 1 μM Ang II, 1 μM Ang II was applied to the ensuing assays.

### 3.2 PON2 inhibited oxidation and inflammatory damage in Ang II‐induced AC16 cells

**Figure 2:**
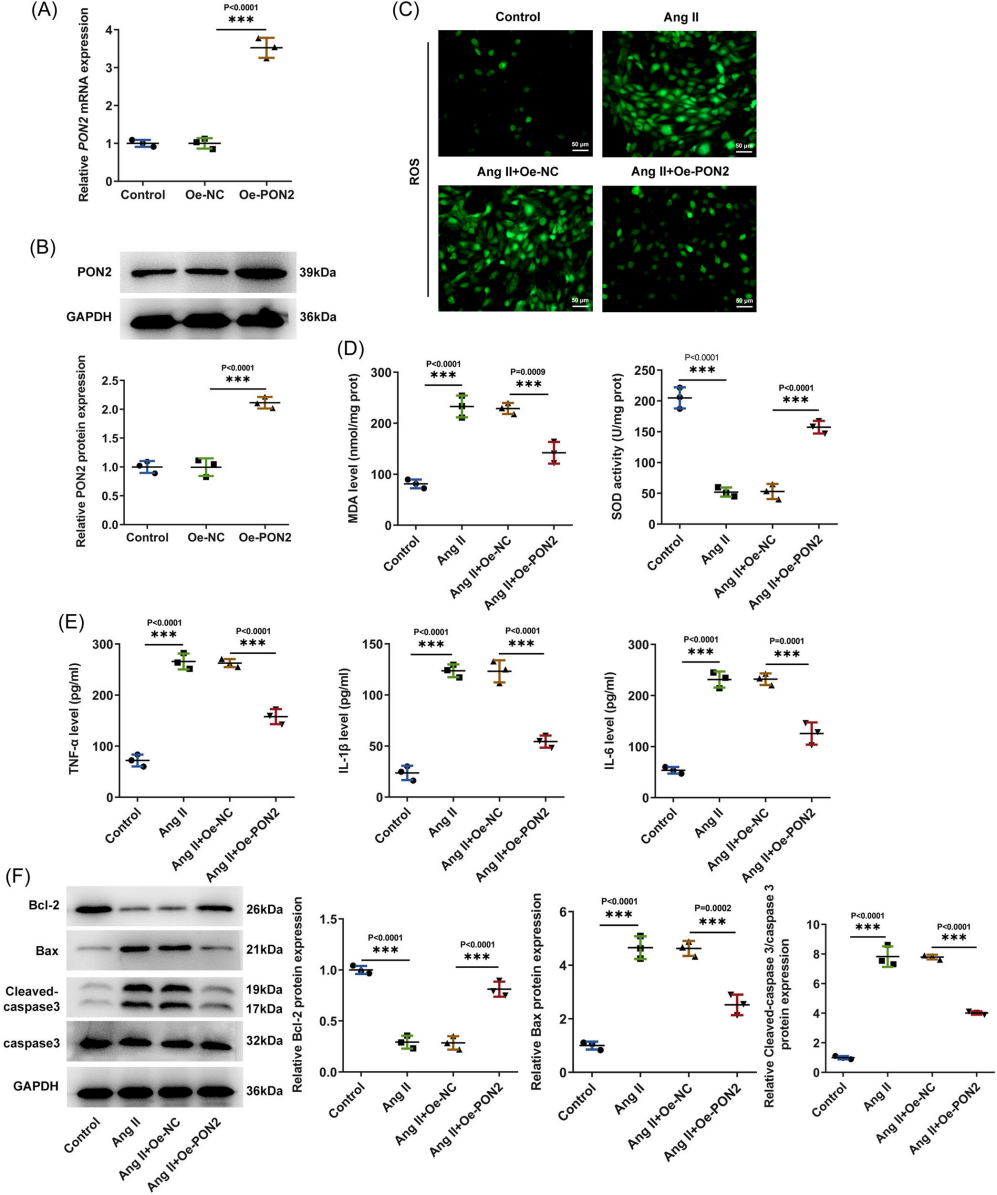
*PON2* inhibited oxidation and inflammatory damage in Ang II‐induced AC16 cells. *PON2* was overexpressed by transfection technique, and RT‐qPCR (A) and Western blot (B) tested transduction efficacy. (C) DCFH‐DA assay appraised ROS level. (D) Related kits examined oxidative stress levels. (E) ELISA tested inflammatory levels. (F) Western blot tested the expression of apoptosis‐associated factors. Ang II, angiotensin II; ELISA, enzyme‐linked immunosorbent assay; PON2, paraoxonase 2; ROS, reactive oxygen species; RT‐qPCR, reverse transcriptase‐quantitative polymerase chain reaction.

PON2 was overexpressed by transfection technique, and Western blot and RT‐qPCR tested the transduction efficacy (Figure 2A,B). Subsequently, Control, Ang II, Ang II + Oe‐NC, and Ang II + Oe‐*PON2* groups were assigned. Western blot and RT‐qPCR tested the transduction efficacy (Figure 2C). Oxidative stress levels were examined with related kits. It was noticed that MDA expression was remarkably fortified and SOD expression was notably lessened in AC16 cells after Ang II induction. Overexpression of *PON2* could reverse this trend (Figure 2D). Tumor necrosis factor (TNF)‐α, interleukin (IL)‐1β, and IL‐6 expression were distinctly augmented in Ang II‐treated cells by contrast with the control group. TNF‐α, IL‐1β, and IL‐6 expression in Ang II + Oe‐*PON2* group were conspicuously downregulated relative to Ang II + Oe‐NC group (Figure 2E). Western blot results showed that the expression of Bax and cleaved‐caspase 3 was significantly increased and the expression of Bcl‐2 was significantly decreased in AC16 cells after Ang II induction. Overexpression of *PON2* could reverse this trend (Figure 2F).

### 3.3 *PON2* mitigated Ang II‐elicited hypertrophy in AC16 cells

**Figure 3:**
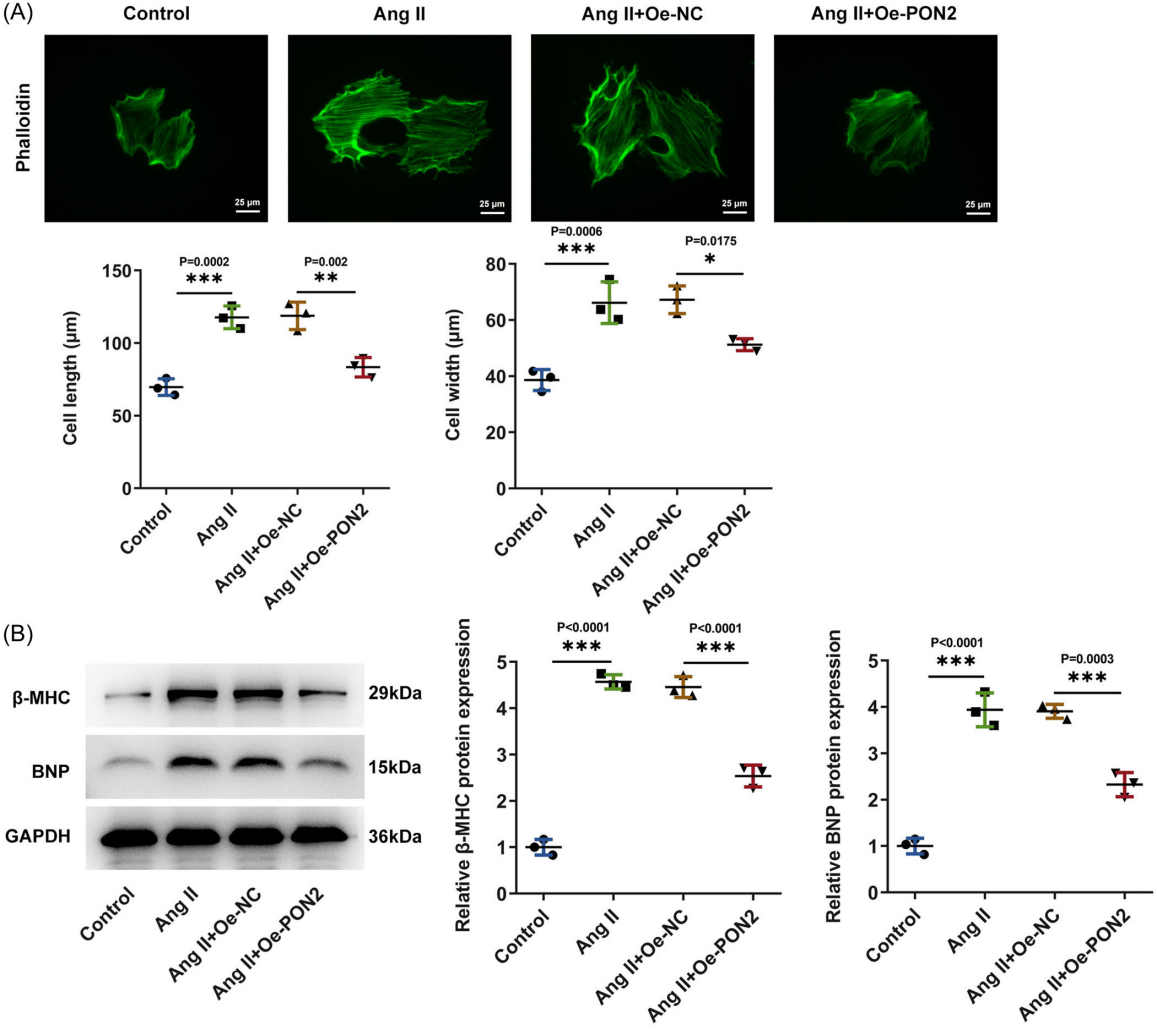
*PON2* mitigated Ang II‐elicited hypertrophy in AC16 cells. (A) Representative images of IF staining for phalloidin following treatment. (B) Western blot tested the expression of hypertrophic markers. Ang II, angiotensin II; IF, immunofluorescence.

Research on cytoskeleton was conducted by IF. The results displayed that cell length was evidently increased in the Ang II group relative to the control group. By contrast with Ang II + Oe‐NC group, cell length in Ang II + Oe‐*PON2* group was apparently reduced (Figure 3A). Western blot analyzed that β‐major histocompatibility complex (β‐MHC), brain natriuretic peptide (BNP) expressions were overtly augmented in AC16 cells after Ang II induction. Following *PON2* elevation, the increase of Ang II induced hypertrophy markers was reversed (Figure 3B).

### 3.4 *PON2* inactivated *CANX*/*NOX4* signaling

**Figure 4:**
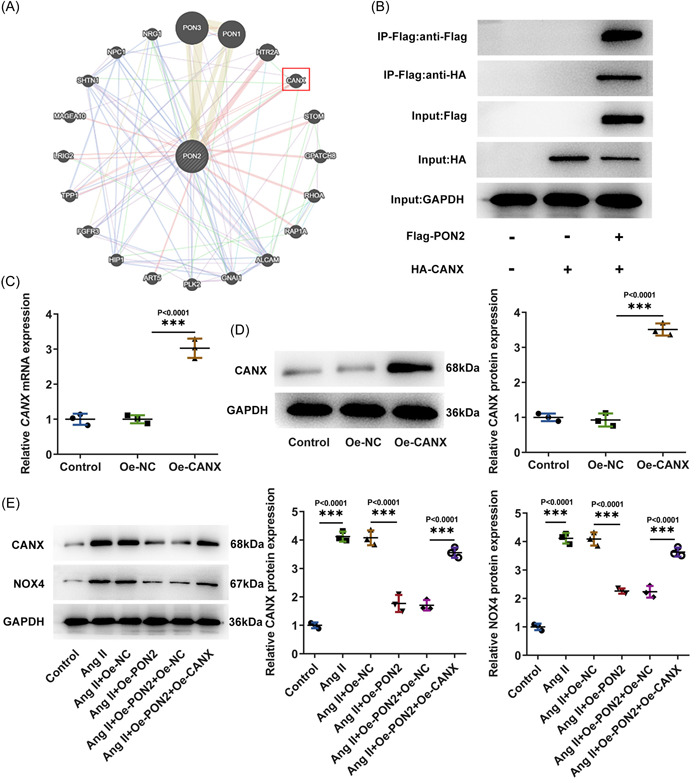
*PON2* inactivated *CANX*/*NOX4* signaling. (A) BioGRID and GeneMania databases were used to analyze the possible interaction between *PON2* and *CANX*. (B) IP assay substantiated the affinity of *PON2* with *CANX*. The overexpression vector of *CANX* was constructed and the vector efficiency was detected by RT‐qPCR (C) and Western blot (D). (E) Western blot tested *NOX4* and *CANX* expression. Ang II, angiotensin II; CANX, Calnexin; IP, immunoprecipitation; PON2, paraoxonase 2; RT‐qPCR, reverse transcriptase‐quantitative polymerase chain reaction.

BioGRID and GeneMania databases were used to analyze the possible interaction between *PON2* and CANX (Figure 4A). IP assay verified the interaction between *PON2* and *CANX* (Figure 4B). Subsequently, the overexpression vector of *CANX* was constructed and the vector efficiency (Figure 4C,D) was detected by RT‐qPCR and Western blot. Moreover, *CANX* and *NOX4* expression were notably elevated after Ang II induction. After *PON2* overexpression, *CANX* and *NOX4* expression were cut down obviously. Relative to Ang II + Oe‐*PON2* + Oe‐NC, *CANX* and *NOX4* expression in Ang II + Oe‐*PON2* + Oe‐*CANX* group were markedly increased (Figure 4E).

### 3.5 *PON2* inhibits oxidation and inflammatory damage in Ang II‐induced AC16 cells by targeting *CANX*/*NOX4* signaling

**Figure 5:**
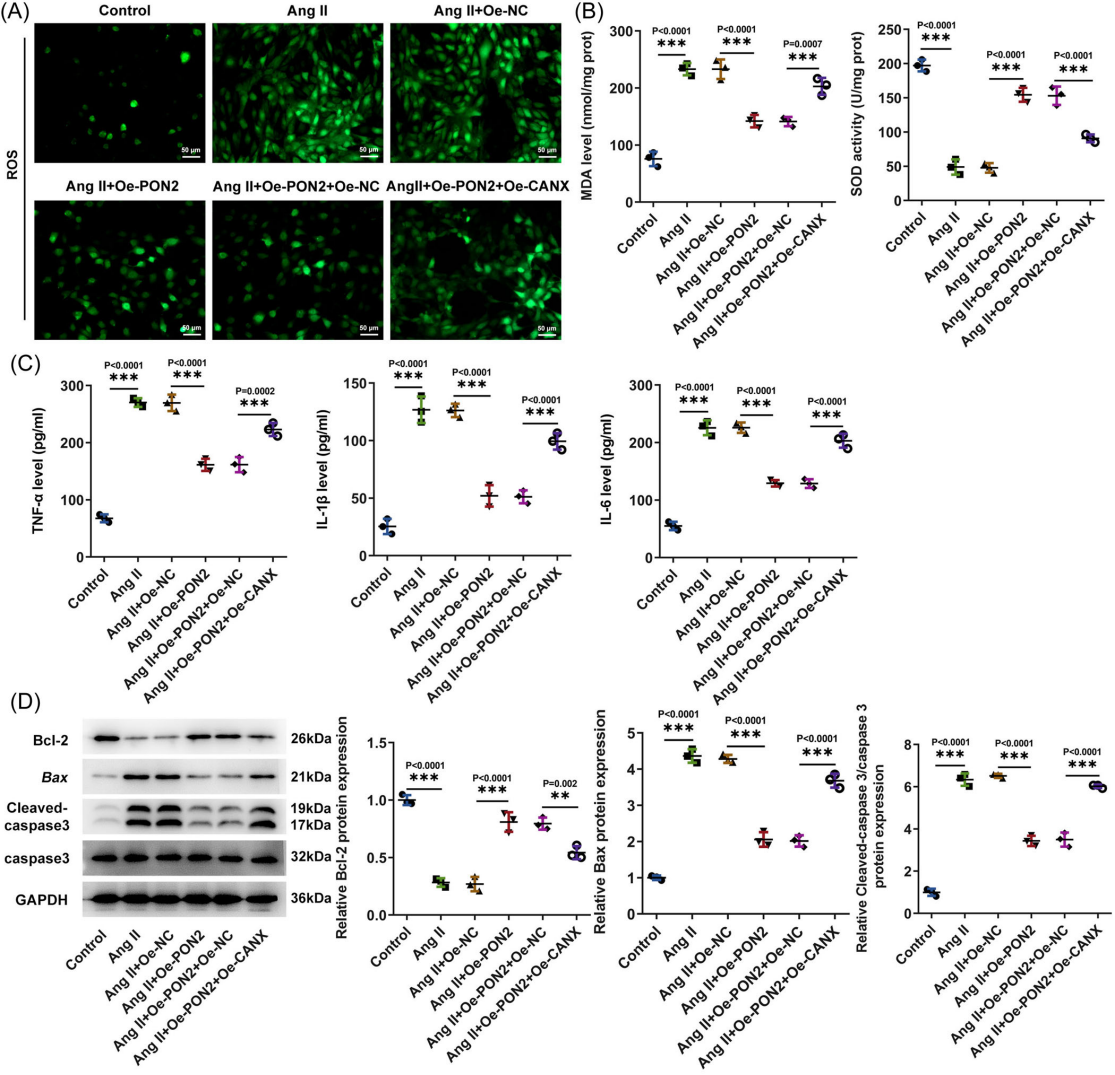
*PON2* inhibited oxidation and inflammatory damage in Ang II‐induced AC16 cells by targeting *CANX*/*NOX4* signaling. (A) DCFH‐DA assay appraised ROS level. (B) Related kits examined oxidative stress levels. (C) ELISA tested inflammatory levels. (D) Western blot tested the expression of apoptosis‐associated factors. Ang II, angiotensin II; CANX, Calnexin; ELISA, enzyme‐linked immunosorbent assay; IP, immunoprecipitation; PON2, paraoxonase 2; RT‐qPCR, reverse transcriptase‐quantitative polymerase chain reaction.

Ccontrol, Ang II, Ang II + Oe‐NC, Ang II + Oe‐*PON2*, Ang II + Oe‐*PON2* + Oe‐NC, and Ang II + Oe‐*PON2* + Oe‐*CANX* groups were assigned. DCFH‐DA assay corroborated that ROS expression in Ang II + Oe‐*PON2* + Oe‐*CANX* group was remarkably higher than that in Ang II + Oe‐*PON2* + Oe‐NC group (Figure 5A). It turned out that relative to Ang II + Oe‐*PON2* + Oe‐NC group, MDA expression in Ang II + Oe‐*PON2* + Oe‐*CANX* group was noticeably raised, while SOD expression was declined (Figure 5B). ELISA results exhibited that TNF‐α, IL‐1β, and IL‐6 expression were significantly increased in Ang II + Oe‐*PON2* + Oe‐*CANX* group compared with Ang II + Oe‐*PON2* + Oe‐NC group (Figure 5C). Western blot uncovered that by contrast with Ang II + Oe‐*PON2* + Oe‐NC group, Bax and cleaved‐caspase 3 expressions were noticeably raised and Bcl‐2 expression was prominently lowered in Ang II + Oe‐*PON2* + Oe‐*CANX* group (Figure 5D).

### 3.6 *PON2* inhibits hypertrophy in Ang II‐induced AC16 cells by targeting *CANX*/*NOX4* signaling

**Figure 6:**
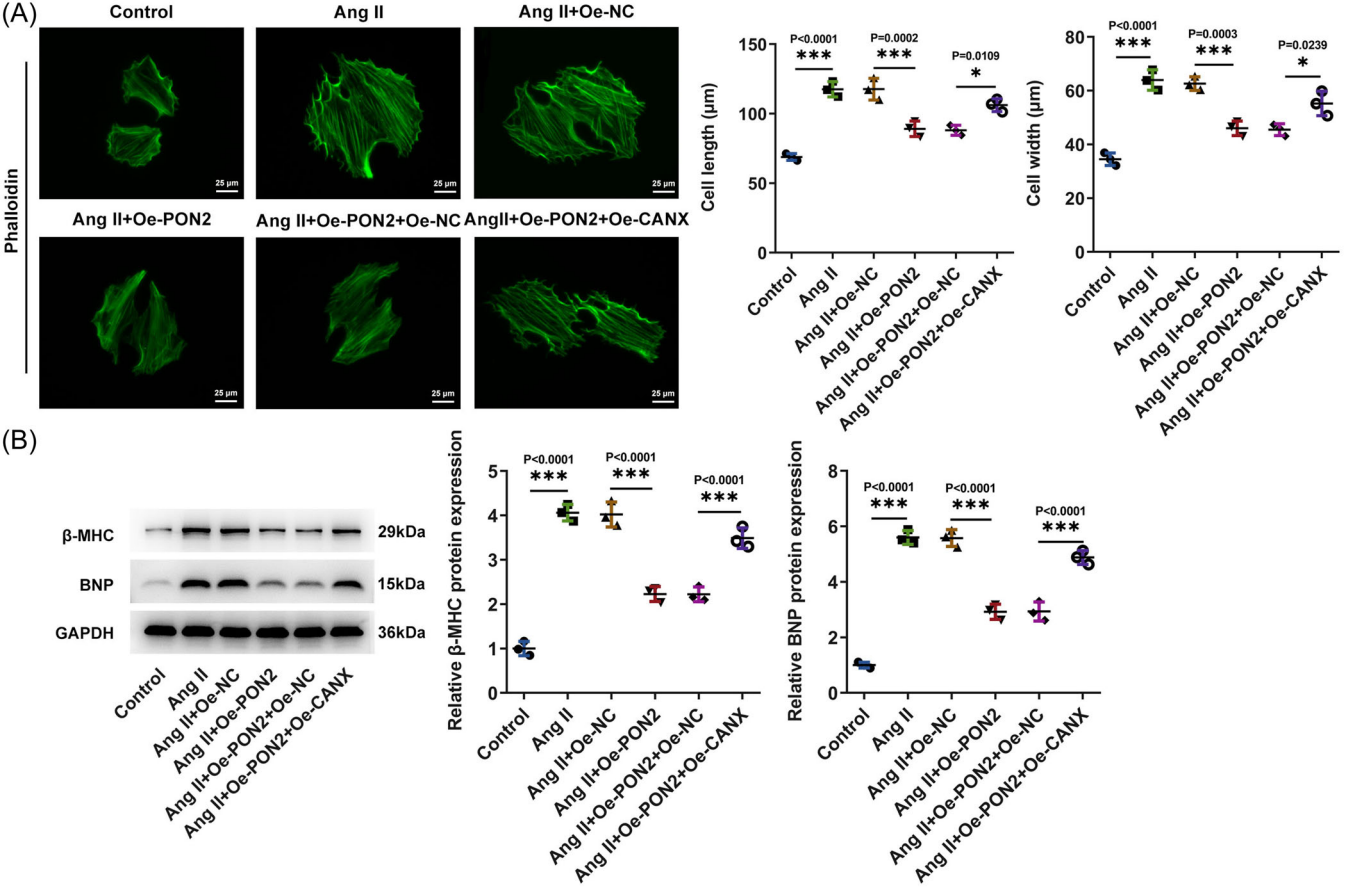
*PON2* inhibited hypertrophy in Ang II‐induced AC16 cells by targeting *CANX*/*NOX4* signaling. (A) Representative images of IF staining for phalloidin following treatment. (B) Western blot tested the expression of hypertrophic markers. Ang II, angiotensin II; CANX, Calnexin; IF, immunofluorescence; PON2, paraoxonase 2.

The cell length of Ang II + Oe‐*PON2* + Oe‐*CANX* group was significantly increased compared with that of Ang II + Oe‐*PON2* + Oe‐NC group (Figure 6A). Western blot unmasked that β‐MHC, and BNP expressions in Ang II + Oe‐*PON2* + Oe‐*CANX* group were distinctly higher than those in Ang II + Oe‐*PON2* + Oe‐NC group (Figure 6B).

## 4 DISCUSSION

At present, the prevalence of HF is on the rise, and long‐term use of diuretics, β‐receptor blockers and other HF drugs is prone to drug resistance. And the prognosis of HF remains unfavorable.^14^ The increasing mortality of HF is still difficult to be effectively controlled, which severely impacts the physical and mental health of patients.^15^ In addition, the etiology of HF remains complicated and diverse, and no clear pathogenesis has been reported. Therefore, it is of urgence to seek for effective therapeutic targets and mechanisms to improve HF.

Under pathological circumstances, excessive collagen deposition occurs in the myocardial interstitium, leading to cardiac fibrosis. Cardiac fibrosis is a common feature of many cardiovascular diseases and ultimately leads to HF.^16^ In HF, decreased cardiac output and insufficient renal perfusion lead to the activation of the RAAS system and the increase of plasma Ang II secretion. Then Ang II binds to angiotensin receptor 1, resulting in cardiac fibroblast proliferation, overexpression of intercellular collagen and matrix deposition, and so on, eventually contributing to myocardial fibrosis.^17^ Therefore, Ang II was used to induce AC16 cells in vitro to form a model of the damage of cardiomyocytes. We found that after Ang II induction, oxidative stress was potentiated, inflammatory response was exacerbated and apoptosis increased, and hypertrophy occurred.

After Ang II treatment, we found that *PON2* expression was declined dramatically in AC16 cells. A previous study has shown that the expression of *PON2* was also significantly reduced in Ang II‐induced vascular smooth muscle cells and hypertensive rat vascular tissues.^9^ In addition, a new antihypertrophy effect of the PON gene cluster provides a possible strategy for treating cardiac hypertrophy by increasing the level of the PON gene family.^18^ *PON2* deficiency significantly exacerbates left ventricular remodeling and cardiac fibrosis after transverse aortic contraction.^6^ Moreover, *PON2* protects against acute myocardial ischemia‐reperfusion injury by regulating mitochondrial function and oxidative stress through the PI3K/Akt/GSK‐3β RISK pathway.^5^ These findings hint that *PON2* acts as a suppressor in HF‐related cardiac diseases. Subsequently, we overexpressed the expression of *PON2* in AC16 cells induced by Ang II and found that after overexpression of *PON2*, oxidative stress was alleviated, inflammation was diminished, cell apoptosis was obstructed, and the trend of hypertrophy was reversed. As one of the pivotal pathological alternations in the initiation and process of chronic HF, myocardial hypertrophy is mainly featured by collagen fiber hyperplasia and myocardial hypertrophy.^19^ Thereafter, the severity of HF was investigated in our experiment by detecting the degree of myocardial hypertrophy of AC16 cells.

Next, the regulatory mechanism of *PON2* was further delved into. We analyzed the genes interacting with *PON2* using BioGrid and GeneMania databases and found that *PON2* may target *CANX*. We verified the interaction between *PON2* and *CANX* through IP experiments. It has found that *CANX* expression is upregulated in the rat model of transverse aortic coarctation.^20^ *CANX* is involved in the development of arrhythmia through oocyte meiosis and focal expression and is considered as a potential biomarker of arrhythmia.^21^ These findings point out that *CANX* exert vital properties on regulating heart disease. In addition, *CANX* is a *NOX4* interaction protein, and lowering *CANX* can reduce *NOX4* expression and reactive oxygen species formation.^11^ Meanwhile, study has shown that *PON2* overexpression can inhibit *NOX4* expression level.^12^ Therefore, it is reasonable to speculate that *PON2* reduces Ang II‐induced cardiomyocyte injury through targeted inhibition of *CANX*/*NOX4* signaling. In the experiment, *PON2* and *CANX* expression in AC16 cells were elevated concurrently, and found that *PON2* reduced the myocardial cell damage caused by Ang II by targeting the inhibition of *CANX*/*NOX4* signal pathway.

There are some limitations to this article. First of all, we did not conduct experiments on primary cardiomyocytes, but chose AC16 cell lines with cardiomyocyte characteristics. In future experiments, we will further verify our conclusions in primary cardiomyocytes. Second, we did not further explore the mechanism at the animal level, and our future experiments will further explore the mechanism in animals.

## 5 CONCLUSION

In this study, we found that *PON2* ameliorates Ang II‐induced cardiomyocyte injury by targeting the *CANX*/*NOX4* signaling pathway. Our paper provides a theoretical basis for the treatment of HF.

## AUTHOR CONTRIBUTIONS

Baopeng Tang and Ping Fan contributed to the conception and design of the present study, analyzed and interpreted the data, and critically revised the manuscript for important intellectual content. Yuanzheng Ye, Jian Zhang and Yankai Guo contributed to designing the study, and analyzed the data. Yuanzheng Ye and Jiajun Zhu drafted and revised the manuscript. Baopeng Tang and Ping Fan confirm the authenticity of all the raw data. The final manuscript has been read and approved by all authors.

## CONFLICT OF INTEREST STATEMENT

The authors declare no conflict of interest.

## Data Availability

The datasets used and/or analyzed generated during the current study are available from the corresponding author on reasonable request.
